# Elderly out-of-hospital cardiac arrest has worse outcomes with a family bystander than a non-family bystander

**DOI:** 10.1186/1865-1380-5-41

**Published:** 2012-11-09

**Authors:** Manabu Akahane, Seizan Tanabe, Soichi Koike, Toshio Ogawa, Hiromasa Horiguchi, Hideo Yasunaga, Tomoaki Imamura

**Affiliations:** 1 Department of Public Health, Health Management and Policy, Nara Medical University School of Medicine, 840 Shijo-cho, Kashihara, Nara, 634-8521, Japan; 2 Foundation for Ambulance Service Development, Emergency Life-Saving Technique Academy of Tokyo, Tokyo, Japan; 3 Department of Planning, Information and Management, The University of Tokyo Hospital, Tokyo, Japan; 4 Department of Health Management and Policy, Graduate School of Medicine, The University of Tokyo, Tokyo, Japan

**Keywords:** Out-of-hospital cardiac arrest, Elderly, Bystander cardiopulmonary resuscitation, Bystander type, Survival rate

## Abstract

**Background:**

A growing elderly population along with advances in equipment and approaches for pre-hospital resuscitation necessitates up-to-date information when developing policies to improve elderly out-of-hospital cardiac arrest (OHCA) outcomes. We examined the effects of bystander type (family or non-family) intervention on 1-month outcomes of witnessed elderly OHCA patients.

**Methods:**

Data from a total of 85,588 witnessed OHCA events in patients aged ≥65 years, which occurred from 2005 to 2008, were obtained from a nationwide population-based database. Patients were stratified into three age categories (65–74, 75–84, ≥85 years), and the effects of bystander type (family or non-family) on initial cardiac rhythm, rate of bystander cardiopulmonary resuscitation (CPR), and 1-month outcomes were assessed.

**Results:**

The overall survival rate was 6.9% (65–74 years: 9.8%, 75–84 years: 6.9%, ≥85 years: 4.6%). Initial VF/VT was recorded in 11.1% of cases with a family bystander and 12.9% of cases with a non-family bystander. The rate of bystander CPR was constant across the age categories in patients with a family bystander and increased with advancing age categories in patients with a non-family bystander. Patients having a non-family bystander were associated with significantly higher 1-month rates of survival (OR: 1.26; 95% CI: 1.19–1.33) and favorable neurological status (OR: 1.47; 95% CI: 1.34–1.60).

**Conclusions:**

Elderly patient OHCA events witnessed by a family bystander were associated with worse 1-month outcomes than those witnessed by a non-family bystander. Healthcare providers should consider targeting potential family bystanders for CPR education to increase the rate and quality of bystander CPR.

## Background

Out-of-hospital cardiac arrest (OHCA) is one of the most common causes of death in middle and old age, and it is an important public health concern. OHCA is associated with a high mortality rate [1], even when patients receive appropriate treatment in accordance with the links in the “chain of survival” concept, consisting of rapid access to emergency medical services (EMS), cardiopulmonary resuscitation (CPR), defibrillation including public-access automated external defibrillation (PAD), and advanced cardiovascular life support (ACLS) [2-6].

Early bystander CPR can be critical in improving the survival rate and neurological outcome after OHCA [2,3,5-7]. The outcome of cardiopulmonary arrest, as measured by survival rate and cerebral performance category (CPC), is worse when the quality of CPR is suboptimal in some way, such as insufficient depth of chest compression or an excessive number of ventilations. Poor OHCA outcomes may therefore be partly due to delay in starting CPR or suboptimal CPR quality [7]. A better understanding of the reasons why bystander CPR is not performed in some elderly OHCA patients may assist in finding ways to increase the rate of bystander CPR and thereby improve the outcome.

A patient receiving high-quality, early CPR has a better chance of survival with intact neurological status, indicating that the quality and timing of bystander CPR can have a significant impact on outcome. The quality and the timing of bystander CPR might be affected by bystander characteristics such as age, gender, and bystander type (family or non-family). Approximately 70% of OHCA events occur at home [8-10]. In elderly OHCA patients, a family member such the patient’s spouse may be the bystander performing CPR, and the spouse may be an elderly person with physical limitations, which may affect the quality of CPR performed and the speed of calling the EMS or starting bystander CPR.

In this study, we describe the characteristics and outcomes of elderly OHCA cases using a nationwide, population-based database and evaluate the impact of bystander type (family or non-family) on outcomes. The age of the OHCA patient may affect the response, such as affecting ACLS administration by EMS in the ambulance and treatment by physicians after arrival at the hospital. This study can provide important information to support developing policies to increase the rate of bystander CPR and improve outcomes when resuscitating elderly OHCA patients.

## Methods

### Study design and data sources

This is an observational study using a database of all recorded OHCA patients transported to the hospital in Japan from January 2005 to December 2008. The Fire and Disaster Management Agency (FDMA) administers the EMS in Japan and provides the only ambulance service, which is available to all citizens. Japan has 807 local fire departments with dispatch centers, and all OHCA patients transported to the hospital by EMS were recorded in a national OHCA database by the FDMA. EMS personnel gathered the data, and the database was maintained by the local fire departments. All data were verified and anonymized at the local fire department; they were then transferred to and stored in the national-level OHCA database developed by the FDMA for public use. With permission from the FDMA, we extracted data from the national database to perform a nationwide, population-based study using all recorded cases of OHCA in Japan over a 4-year period. The Ethics Committee of Nara Medical University approved the study design (authorization code: 260).

### Patient details and outcomes

The OHCA data entry form was largely based on the Utstein form [11] and was extended to include details of all OHCA cases, including those due to non-cardiac causes such as stroke, asphyxia, and trauma, as well as unwitnessed cases [12-15]. Patient data collected included age, sex, initial cardiac rhythm, whether the OHCA was witnessed by a bystander or not, whether bystander CPR was performed or not, bystander category (family, layperson other than family, or EMS personnel), the time of collapse, call to EMS, start of bystander CPR, whether or not EMS attempted defibrillation, and the outcome in terms of survival and CPC at 1 month after OHCA. Nursing home staff members were classified as laypersons other than family in this study. Initial cardiac rhythm was categorized by EMS as ventricular fibrillation (VF), pulseless ventricular tachycardia (VT), pulseless electrical activity, or asystole.

EMS personnel collected data regarding survival and neurological status at 1 month from the hospitals that had received the patients, in cooperation with the physicians who had treated the patients. Neurological status at 1 month was determined by assessing CPC by follow-up interview.

### Subjects

During the 4-year study period, data from 431,950 OHCA patients were included in the national database. To determine differences due to bystander type in witnessed elderly OHCAs, we selected witnessed OHCA patients aged ≥65 years with a time interval from the call to EMS to EMS arrival at the scene of ≤60 min and excluded cases with an EMS personnel bystander. We stratified cases into three age categories as follows: 65–74 years, 75–84 years, and ≥85 years. In total, data from 85,588 cases of witnessed elderly OHCA events were analyzed.

### Data analysis

To assess whether bystander type affected outcomes in witnessed elderly OHCAs, we divided cases into two groups based on bystander type (family or non-family) and analyzed whether bystander CPR was performed, whether there was initial VF/VT, and whether PAD was performed. We also determined the time intervals (minutes) from the call to EMS to EMS arrival at the scene, from collapse to the start of bystander CPR, and from collapse to the call to EMS. Analyses of the time intervals from collapse to the start of bystander CPR and from collapse to the call to EMS were limited to those with an interval of ≤60 min. We measured outcomes in terms of 1-month survival and favorable neurological status at 1 month in the three age categories. A favorable neurological status was defined as CPC category 1 (good cerebral performance) or 2 (moderate cerebral disability) [16].

Data were examined by bystander type and age category. Statistical analyses were conducted to examine differences between groups in terms of the factors mentioned above using a *t*-test or chi-square test as appropriate. Logistic regression analyses were performed to identify the effects of bystander type on 1-month outcomes (survival and favorable neurological status), using family bystander as a reference. Potential confounding factors included age category, sex, bystander type, bystander CPR, attempted defibrillation by EMS, use of PAD, and interval from the call to EMS to EMS arrival at the scene. In this analysis, bystander CPR included chest compression only, mouth-to-mouth ventilation only, and chest compression with mouth-to-mouth ventilation (conventional CPR).

## Results

Males accounted for 56.6% of elderly OHCA patients. The mean age of males (78.5 years) was significantly younger than that of females (83.0 years) (*P* < 0.001). The overall 1-month survival rate was 6.9% and of favorable neurological status was 2.8%. Table 1 shows the 1-month outcomes by initial cardiac rhythm, bystander CPR type, whether or not PAD was performed, age category, and bystander type. The 1-month outcomes of OHCA with chest compression only and with conventional CPR were very similar. The 1-month rates of survival and favorable neurological status decreased with increasing age categories.

**Table 1:** Overall 1-month outcomes for witnessed elderly OHCA patients

	***n***	**1-month outcomes**			
		**Survival**		**Favorable CPC**	
		***n***	**%**	***n***	**%**
Initial cardiac rhythm					
VF/VT	9,977	1,972	19.8	1,066	10.7
PEA	29,652	1,855	6.3	493	1.7
Asystole	43,319	1,177	2.7	186	0.4
Bystander CPR					
Non	48,263	2,981	6.2	1,024	2.1
Chest compression only	20,283	1,569	7.7	746	3.7
M-to-M ventilation only	1,160	84	7.2	38	3.3
Conventional CPR	15,880	1,291	8.1	594	3.8
PAD					
Not performed	82,614	5,639	6.8	2,245	2.7
Performed	480	118	24.6	94	19.6
Age category					
65–74 years	23,338	2,291	9.8	1,117	4.8
75–84 years	33,832	2,327	6.9	845	2.5
85 years and over	28,416	1,307	4.6	440	1.6
Bystander type					
Family	57,563	3,548	6.2	1,292	2.2
Non-family	28,023	2,377	8.5	1,110	4.0

OHCA: out-of-hospital cardiac arrest, CPC: cerebral performance categories, CPR: cardiopulmonary resuscitation, VF: ventricular fibrillation, VT: pulseless ventricular tachycardia, PEA: pulseless electrical activity, M-to-M: mouth-to-mouth, PAD: public access automated external defibrillator.

Table 2 shows basic demographic characteristics by bystander type. Bystander CPR rate was 43.6% overall, 35.5% in cases with a family bystander, and 60.2% in cases with a non-family bystander. Cases with a non-family bystander had a significantly higher rate of bystander CPR, including both chest compression only (26.4% vs. 22.4% with a family bystander) and conventional CPR (32.5% vs. 11.8% with a family bystander). The rates of PAD and initial VF/VT were higher among patients with a non-family bystander than those with a family bystander. The 1-month outcomes were also significantly better in the non-family bystander group. The intervals from collapse to start of bystander CPR and from collapse to the call to EMS were significantly shorter among cases with a non-family bystander than those with a family bystander.

**Table 2:** Demographic characteristics and outcomes by bystander type

	**Bystander type**				***P *value**
	**Family (*n *= 57,565)**		**Non-family (*n *= 28,023)**		
	***n***	**%**	***n***	**%**	
Age category					
65–74 years	15,854	27.5	7,485	26.7%	0.011
75–84 years	24,032	41.7	9,800	35.0%	<0.001
85 years and over	17,679	30.7	10,738	38.3%	<0.001
Initial cardiac rhythm					
VF/VT	6,361	11.1	3,616	12.9%	<0.001
PAD performed	72	0.1	408	1.5%	<0.001
Bystander performed CPR					
Non	37,112	64.5	11,152	39.8%	<0.001
Chest compression only	12,881	22.4	7,402	26.4%	<0.001
M-to-M ventilation only	792	1.4	368	1.3%	0.468
Conventional CPR	6,780	11.8	9,101	32.5%	<0.001
1-Month outcomes					
Survival	3,548	6.2	2,377	8.5%	<0.001
Favorable CPC	1,292	2.2	1,110	4.0%	<0.001
Interval	Min	SD	Min	SD	
Call–arrival of EMS	7.5	3.9	7.1	3.7	<0.001
Collapse–bystander CPR	4.8	6.9	2.0	4.2	<0.001
Collapse–call	5.6	7.8	4.9	6.6	<0.001

EMS: emergency medical service; other abbreviations as for Table 1.

**Figure 1:**
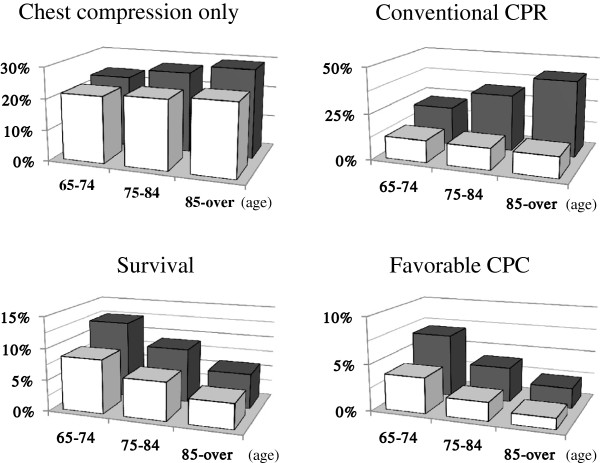
**Rates of bystander cardiopulmonary resuscitation (CPR) and 1-month outcomes by bystander type and age category.** White columns indicate cases with a family bystander, and gray columns indicate cases with a non-family bystander. Numbers on the horizontal axes indicate age categories.

Figure 1 shows the rates of bystander CPR and 1-month outcomes by bystander type and age category. Interestingly, the rate of bystander CPR was constant among age categories in cases with a family bystander (white column in Figure 1), but increased with advancing age categories among patients with a non-family bystander (gray column). The rate of conventional CPR was markedly higher among patients with a non-family bystander.

Table 3 shows the results of logistic regression analyses for outcomes at 1 month. Cases with a non-family bystander had significantly higher rates of 1-month survival (OR: 1.26; 95% CI: 1.19–1.33) and favorable neurological status (OR: 1.47; 95% CI: 1.34–1.60). CPR consisting of either chest compression only or conventional CPR was associated with higher rates of 1-month survival and favorable neurological status. PAD was also associated with a significantly higher 1-month rate of survival and of favorable neurological status.

**Table 3:** Results of logistic regression analyses for 1-month outcomes

	**1-Month outcomes**					
	**Survival**			**Favorable CPC**		
	**OR**	**95% CI**	***P *value**	**OR**	**95% CI**	***P *value**
Bystander type						
Family	Reference			Reference		
Non-family	1.26	1.19-1.33	<0.001	1.47	1.34-1.60	<0.001
Sex						
Male	Reference			Reference		
Female	0.95	0.89-1.01	0.076	0.96	0.88-1.05	0.373
Age category						
65–74 years	Reference			Reference		
75–84 years	0.79	0.75-0.85	<0.001	0.63	0.58-0.70	<0.001
85 years and over	0.54	0.50-0.58	<0.001	0.40	0.36-0.46	<0.001
Bystander CPR						
Non	Reference			Reference		
Chest compression only	1.34	1.25-1.43	<0.001	1.84	1.67-2.04	<0.001
M-to-M ventilation only	1.21	0.97-1.53	0.098	1.62	1.16-2.27	0.005
Conventional CPR	1.39	1.29-1.50	<0.001	1.78	1.59-2.00	<0.001
PAD						
Not performed	Reference			Reference		
Performed	2.87	2.29-3.60	<0.001	4.55	3.53-5.86	<0.001
Defibrillation by EMS						
Not performed	Reference			Reference		
Performed	2.91	2.74-3.09	<0.001	3.88	3.55-4.23	<0.001
Call−arrival of EMS interval (1-min increase)	0.89	0.88-0.90	<0.001	0.86	0.85-0.88	<0.001

OR: odds ratio, CI: confidence interval; other abbreviations as for Table 2.

## Discussion

In this study we assessed the effect of bystander type on 1-month outcomes (survival and favorable neurological status) among elderly OHCA patients. Our results revealed that OHCA patients with a non-family bystander were more likely to survive compared than those with a family bystander. The rate of bystander CPR increased with advancing age categories among patients with a non-family bystander, but seemed to be the same across all age categories in cases with a family bystander. Bystander chest compression only and conventional CPR had significant impacts and similar 1-month outcomes, and PAD also had a significant impact on 1-month outcomes.

Many previous reports have described outcomes among elderly patients with cardiopulmonary arrest [17-21]. However, most studies were published in the 1980s and 1990s and had relatively small data sets. Over the past few decades, countries with increased life expectancies are having larger numbers of elderly people. The resuscitation techniques and equipment used by pre-hospital EMS and hospital physicians are advancing, and it is therefore important for these care providers to have up-to-date information about the characteristics and outcomes of OHCA cases among the elderly. Since some characteristics of OHCA patients such as their age may affect the decisions made by EMS regarding the provision of ACLS and the treatment decisions made by physicians after hospital arrival, the present study provides important information to support correct decision-making by EMS and physicians. Up-to-date information is an important consideration when developing effective policies to increase bystander CPR rates and improve outcomes.

Previous studies have reported that approximately half of OHCA patients did not receive bystander CPR before EMS arrival at the scene [9,22]. Hauff *et al.*[22] reported that the physical limitations of bystanders were the major reason for lack of bystander CPR, even when a dispatcher provided CPR instructions via telephone. Lack of bystander CPR often appeared to be due to a combination of the bystander’s physical limitations and the position of the OHCA patient. Patient emesis and bystander concerns about disease transmission also appeared to impede bystander CPR.

Recently, several papers have reported on the outcomes of elderly OHCA patients [23,24]. Deasy *et al.*[23] studied 30,006 OHCA patients attended by paramedics, of whom 32% were aged 65–79 years, 21% were 80–89 years, 5% were 90–99 years, and 0.1% were ≥100 years. The rate of attempted resuscitation decreased with advancing age, with overall survival rates to hospital discharge of patients aged 65–79 years, 80–89 years, and 90–99 years of 8%, 4%, and 2%, respectively. They also assessed information about the location of collapse and reported that the proportion of OHCA events occurring at nursing homes increased with advancing age. By comparison, the present study indicated a slightly higher survival rate, with an overall bystander CPR rate of <50% and an increasing bystander CPR rate with advancing age categories among non-family-witnessed OHCA patients. Surprisingly, the rate of bystander CPR was only 35.5% among cases with a family bystander, indicating that OHCA patients with family bystanders were less likely to receive bystander CPR than those with a non-family bystander, especially in the older age categories.

The relatively good 1-month outcomes in the present study could be explained by the selection of only witnessed OHCA cases. Most OHCA cases with a family bystander may occur at the patient’s home, whereas patients in the more advanced age groups may be more likely to be in a nursing home where OHCA could be witnessed by nursing home staff, which would increase the proportions of cases with a non-family bystander. Nursing home staff classified as non-family bystanders may have basic life support (BLS) training and may be accustomed to dealing with OHCA, resulting in a higher bystander CPR rate and earlier performance of bystander CPR, which could achieve a higher initial VF/VT rate and better outcomes. It is known that immediate bystander CPR maintains VF longer in OHCA patients, which is a strong predictor of survival [25]. Our results also indicate a shorter interval from the call to EMS to EMS arrival at the scene and from collapse to the call to EMS in the non-family bystander group compared to the family bystander group, which could also affect the rate of initial VF/VT and 1-month outcomes. As the difference in the interval from collapse to bystander CPR was more marked than the differences in intervals from the call to EMS to EMS arrival or from collapse to the call to EMS between the family and non-family groups, the interval from collapse to bystander CPR seemed to have the most impact on initial VF/VT rates and 1-month outcomes. Family bystanders may be elderly people such as the spouse of the OHCA patient and may have physical limitations that make it difficult to perform bystander CPR compared to a younger non-family bystander such as a colleague, passer-by, or facility staff member.

It has been reported that patients with known heart disease received bystander CPR in only 16% of cases [26] and that older people are not very willing to learn CPR even when they have a family member with known heart disease [27]. Generally, a large proportion of OHCA events occurs at the patient’s home, and these have a poor prognosis [9,28]. Herlitz *et al.*[9] reported the characteristics and outcomes of OHCA patients who collapsed at home compared with those who collapsed in other places. Those who collapsed at home were witnessed less often, received bystander CPR less often, were found to have VF less often, and had a longer interval between collapse and call to EMS, start of CPR, and first defibrillation. Furthermore, conventional bystander CPR (chest compression with ventilation) was performed less frequently when the collapse was in the patient’s home. While they concluded that OHCA occurring at home was a strong independent predictor of adverse outcome, they did not give reasons for this. Even though they identified the bystander as layperson, ambulance personnel, medical personnel, or police, they did not distinguish if a layperson was a family or non-family bystander. Jackson *et al.*[8] reported that OHCA occurring outside the home was associated with improved outcomes. Patients with witnessed OHCA outside the home were more likely to receive bystander CPR and to survive. Our results showed a lower bystander CPR rate and a higher rate of adverse outcomes in cases with a family bystander compared to a non-family bystander.

It has been suggested that simplifying the CPR technique to include chest compression only may increase the rate of bystander CPR in elderly OHCA patients when a dispatcher provides telephone CPR instruction [29]. In the present study, both bystander chest compression only and bystander conventional CPR were associated with improved outcomes, with both having a similar level of impact on the rates of 1-month survival and favorable neurological status. The simpler procedure of chest compression only might therefore be appropriate for dispatcher-assisted telephone CPR for elderly OHCA patients when the bystander is an elderly person with physical limitations or emotional distress.

### Limitations

Several limitations of our study should be acknowledged. First, the database did not include detailed information about bystanders such as age and gender, the quality of bystander CPR, and whether the bystander had BLS or ACLS training. Therefore, we could not assess the influence of these factors on outcome. Second, PAD was implemented because early defibrillation using PAD has a significant impact on survival and favorable neurological outcome. Therefore, the accessibility of PAD in OHCA patients with family versus non-family bystanders should be evaluated. However, we could not assess the effects of OHCA location because the database did not include this information. The type of family bystander may be different depending on whether the OHCA occurs at home or elsewhere. Third, we did not have data regarding the medical histories or comorbidities of OHCA patients. Fourth, there is a possibility that family members may have been aware of patient preferences not to attempt resuscitation in the event of OHCA. However, the database did not include detailed information about this point.

## Conclusion

Elderly OHCA patients had worse 1-month outcomes when witnessed by a family bystander compared with a non-family bystander. Healthcare providers should consider targeting potential bystanders for CPR education to increase the rate and quality of bystander CPR and to improve the rates of survival and favorable neurological outcome among elderly OHCA patients. In elderly OHCA patients, there may be a need to change scripted instructions from the emergency telephone service to direct the bystander to start chest compressions immediately without ventilation.

## Competing interests

The authors declare that they have no competing interests.

## Authors’ contributions

The contribution of each author was as follows: MA and TI designed this study and conducted data analysis. TO conducted data cleaning. TO, SK, ST, HH, and HY jointly interpreted the results. All authors read and approved the final manuscript.
